# Outcomes of obstructed abdominal wall hernia: results from the UK national small bowel obstruction audit

**DOI:** 10.1002/bjs5.50315

**Published:** 2020-07-10

**Authors:** National Audit of Small Bowel Obstruction Steering Group and National Audit of Small Bowel Obstruction Collaborators, NASBO Steering Group, Matthew J Lee, Thomas M Drake, Adele E Sayers, Ciaran J Walsh, Michael M Davies, Nicola S Fearnhead, John Abercrombie, Austin Acheson, Derek Alderson, Iain Anderson, Simon Bach, Michael Davies, Zaed Hamady, Daniel Hind, Marianne Hollyman, Sarah Hare, Ellen Lee, John Northover, Christopher Lewis, Paul Marriott, Nick Maynard, David Murray, Gillian Tierney, Azmina Verjee, Jonathan Wild, NASBO Collaborators, S Abbott, Y Abdulaal, S Afshar, J Ah‐Chuen, T Ahmed, M Akhtar, F Akram, E Aldred, A Ali, M Aly, A Amajuoyi, V Amin, D Anderson, O Anderson, A Andreou, A Ansari, S Appleton, R Ardley, F Arshad, O Ashour, A Asour, A Athem, M Athersmith, F Ayoub, H Azeem, B Azhar, T Badenoch, C Baillie, D Bandyopadhyay, J Barker, S Barker, B Barkham, R Baron, J Barrie, E Barry‐Yarrow, G Bashir, N Battersby, G Bazoua, N Behar, S Bellam, C Berger, S Bhandari, S Bhasin, S Biggs, C Bisset, L Blake, N Blencowe, T Boam, A Boddy, C Boereboom, M Bogdan, R Bogle, P Bohra, M Boland, H Bolkan, C Borg, R Boulton, G Bouras, M Boyer, J Boyle, G Branagan, H Brewer, C Briggs, J Broadhurst, E Brown, J Brown, L Brown, O Brown, K Burns, K Butcher, M Butler, B Byrne, L Campbell, C Capper, M Cartmell, T Cash, S Chan, N Chandratreya, J Chapman, S Chapman, A Charalabopoulos, C Cheek, S Chok, W Choong, M Chow, J Chowdhury, P Coe, P Conaghan, G Conn, N Cook, T Cook, S Cooper, J Cornish, D Cotton, C Cox, P Coyne, R Crook, J Crozier, G Cuffolo, P Cunha, N Curtis, J Cutting, K Da Costa, L da Silva, B Das, M Davenport, J Davies, T Davies, A Day, S Dayal, S Dean, G Demetriou, F Dengu, R Dennis, H Dent, P Dent, M Deputy, L Devoto, G Di Benedetto, S Dindyal, E Donnelly, P Doody, E Douka, C Downham, H Dowson, H Edent, K Edgerton, N Ekpete, M El Farran, O Elamin, M Eljaafari, N Elsaid, M El‐Sharif, J Evans, M Evans, R Ewe, A Ewing, K Exarchou, R Fallaize, M Faoury, S Farag, E Farinella, G Faulkner, H Ferguson, O Fisher, J Fletcher, A Forouzanfar, A Foster, R Fox, N Francis, V Fretwell, D Fung, E Gammeri, J Garnham, A Geraghty, A Gilbert, C Gill, M Gill, M Gillespie, P Giordano, J Glasbey, M Goh, A Golder, N Green, T Gregoir, T Grey, E Groundwater, T Grove, S Growcott, S Gunasekaran, H Habib, J Haddow, V Halahakoon, C Halkias, C Hall, A Hampson, L Hancock, T Hanna, J Hannay, A Harikrishnan, R Harries, G Harris, J Hartley, K Harvey, P Hawkin, J Hawkins, R Healy, R Heard, R Heartshorne, S Heller, L Hendra, P Herrod, N Heywood, G Hicks, B Hobson, S Holtham, S Holtham, C Hope, P Hopley, T Hossain, S Hossaini, F Howse, T Hubbard, A Humphreys, H Ikram, M Ioannis, M Iqbal, N Iqbal, R Jain, J Jatania, P Jenkinson, S Jokhan, A Jones, C Jones, L Jones, H Joshi, K Joshi, M Joy, P Jull, G Kakaniaris, G Kakaniaris, R Kallam, E Kane, P Kang, R Kanitkar, S Kauser, F Kazmi, M Kedrzycki, S Kelly, J Kendall, M Khan, T Khan, G King, A Kisiel, C Kitsis, I Kolawole, S Korambayil, S Kosasih, A Kosti, A Kotb, S Kouris, K Kshatriya, S Kumar, G Lafaurie, R Lal, A Lau, T Lazim, T Lazim, A Lazzaro, K Lee, R Lefroy, D Leinhardt, D Leinhardt, H Lennon, K Leong, B Levy, E Lim, J Lim, S Lindley, D Liu, P Lloyd, D Locker, S Lockwood, C Lowe, J Lund, R Lunevicius, A Lunt, S Lutfi, A Luther, S Luwemba, P Mahankali‐Rao, S Mahroof, D Mai, S Majid, A Malik, K Malik, K Mann, S Mansour, N Manu, R Mapara, C Martin, J Martin, R Martin, C Mason, L Massey, J Mathias, P Mathur, K Maude, D McArthur, S McCain, S McCluney, M McFall, B McIlroy, S McKay, N McKinley, A McNair, D McWhirter, P Mekhail, K Mellor, J Merchant, L Merker, D Messenger, A Miles, S Mir, A Mishra, P Mistry, V Miu, M Moat, K Mockford, E Mohamed, I Mohamed, M Mondragon‐Pritchard, N Moore, L Moretti, H Morris, T Morrison, V Morrison‐Jones, J Moss, S Moug, D Mountford, R Moynihan, K Muhammad, D Muldoon‐Smith, J Mulholland, M Mullan, E Murgitroyd, K Murugaiyan, A Myers, I Mykoniatis, G Nana, T Nash, A Nassar, R Newton, C Ng, P Ng, P Ng, K Nguyen, K Nguyen, F Nicholas, M Noor, J Nowers, C Nugent, A Nunn, R Nunn, N Obeid, J O'Callaghan, R O'Hara, O Oke, J Olivier, A O'Neill, S O'Neill, D Osei‐Bordom, L Osgood, S Panagiotopoulos, B Panchasara, R Parks, H Patel, P Patel, R Patel, S Patel, K Pawelec, C Payne, K Pearson, G Perin, I Peristerakis, B Petronio, L Phelan, J Phillips, C Pisaneschi, J Pitt, K Plunkett‐Reed, L Ponchietti, A Pouzi, M Pouzi, A Powell, A Powell‐Chandler, N Pranesh, V Proctor, S Pywell, A Qureshi, N Qureshi, M Rahman, Z Rai, S Ramcharan, K Rangarajan, M Rashid, H Reader, A Rehman, S Rehman, C Rengifo, E Richards, N Richardson, A Robinson, D Robinson, B Rossi, F Rutherford, I Sadien, T Saghir, K Sahnan, G Salahia, J Sarveswaran, M Saunders, B Scott, K Scott, A Seager, S Seal, E Sezen, F Shaban, P Shah, P Shah, M Shahmohammadi, A Shamsiddinova, S Shankar, A Sharpe, V Shatkar, A Sheel, T Shields, M Shinkwin, J Shurmer, A Siddika, S Siddiqui, R Simson, P Sinclair, B Singh, S Singh, J Sivaraj, P Skaife, B Skelly, A Skinner, N Slim, C Smart, N Smart, F Smith, I Smith, R Smith, G Spence, A Sreedhar, J Steinke, L Stevenson, E Stewart‐Parker, M Stott, B Stubbs, B Stubbs, N Stylianides, S Subramonia, M Swinkin, M Swinscoe, N Symons, W Tahir, T Taj, K Takacs, J Tam, K Tan, S Tani, N Tanner, D Tao, M Taylor, B Thava, K Thippeswamy, C Thomas, E Thompson, R Thompson, C Thompson‐Reil, C Thorn, F Tongo, G Toth, A Turnbull, J Turnbull, C Valero, G van Boxel, M Varcada, M Venn, N Ventham, M Venza, D Vimalachandran, I Virlos, T Wade, A Wafi, K Waite, M Walker, N Walker, T Walker, U Walsh, S Wardle, R Warner, J Watfah, N Watson, J Watt, J Watts, J Wayman, C Weegenaar, H West, M West, L Whitehurst, M Whyler, M Wiggans, S Wijeyekoon, G Williams, R Williams, A Williamson, J Williamson, J Wilson, A Winter, L Wolpert, J Wong, E Yeap, T Yeong, S Zaman, B Zappa, D Zosimas, West Midlands Research Collaborative

## Abstract

**Background:**

Abdominal wall hernia is a common surgical condition. Patients may present in an emergency with bowel obstruction, incarceration or strangulation. Small bowel obstruction (SBO) is a serious surgical condition associated with significant morbidity. The aim of this study was to describe current management and outcomes of patients with obstructed hernia in the UK as identified in the National Audit of Small Bowel Obstruction (NASBO).

**Methods:**

NASBO collated data on adults treated for SBO at 131 UK hospitals between January and March 2017. Those with obstruction due to abdominal wall hernia were included in this study. Demographics, co‐morbidity, imaging, operative treatment, and in‐hospital outcomes were recorded. Modelling for factors associated with mortality and complications was undertaken using Cox proportional hazards and multivariable regression modelling.

**Results:**

NASBO included 2341 patients, of whom 415 (17·7 per cent) had SBO due to hernia. Surgery was performed in 312 (75·2 per cent) of the 415 patients; small bowel resection was required in 198 (63·5 per cent) of these operations. Non‐operative management was reported in 35 (54 per cent) of 65 patients with a parastomal hernia and in 34 (32·1 per cent) of 106 patients with an incisional hernia. The in‐hospital mortality rate was 9·4 per cent (39 of 415), and was highest in patients with a groin hernia (11·1 per cent, 17 of 153). Complications were common, including lower respiratory tract infection in 16·3 per cent of patients with a groin hernia. Increased age was associated with an increased risk of death (hazard ratio 1·05, 95 per cent c.i. 1·01 to 1·10; *P* = 0·009) and complications (odds ratio 1·05, 95 per cent c.i. 1·02 to 1·09; *P* = 0·001).

**Conclusion:**

NASBO has highlighted poor outcomes for patients with SBO due to hernia, highlighting the need for quality improvement initiatives in this group.

## Graphical abstract

This study shows that small bowel obstruction due to abdominal wall hernia is common, typically involves a co‐morbid group of patients and has poor outcomes, including high mortality rates.

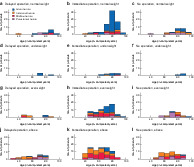

High mortality from this problem

## Abstract (es)

**Antecedentes:**

La eventración es una enfermedad quirúrgica frecuente. Algunos pacientes acuden a urgencias por episodios de obstrucción intestinal, incarceración o estrangulación. La obstrucción del intestino delgado (*small bowel obstruction*, SBO) es una forma de presentación quirúrgica grave asociada a una morbilidad significativa. El objetivo de este estudio fue describir el tratamiento actual y los resultados de los pacientes con obstrucción herniaria en el Reino Unido identificados a través de la *National Audit of Small Bowel Obstruction* (NASBO).

**Métodos:**

La NASBO recopiló datos de los pacientes adultos tratados por obstrucción del intestino delgado en 131 hospitales del Reino Unido entre enero y marzo de 2017. En este estudio se incluyeron solo los que presentaron una obstrucción por una hernia de la pared abdominal. Se registraron los datos demográficos, la comorbilidad, los estudios de imagen, el tratamiento quirúrgico y los resultados hospitalarios. Se realizó una modelización de los factores asociados con la mortalidad y las complicaciones utilizando el análisis de riesgos proporcionales de Cox y modelos de regresión multivariable.

**Resultados:**

De los 2.341 pacientes incluidos en la NASBO, 415 (17,7%) presentaron una SBO por una hernia. La edad media fue de 71,2 (DE 13,9) años, con una comorbilidad moderada (índice de comorbilidad de Charlson 4,6 (DE 7,1)). Fueron operados 312/415 (75,2%). Un total de 198/312 (63,5%) pacientes precisaron una resección del intestino delgado. Se planteó el tratamiento no quirúrgico en 35/65 (53,8%) de las hernias paraestomales y en 38/106 (32,1%) de los pacientes con hernia incisional. La mortalidad hospitalaria fue de 39/415 (9,4%), siendo más elevada en pacientes con hernia inguinal (17/153 (11,1%)). El desarrollo de complicaciones fue habitual, de las que destaca la infección del tracto respiratorio inferior en el 16,3%. El aumento de la edad se asoció con un mayor riesgo de mortalidad (cociente de riesgos instantáneos, *hazard ratio*, HR 1,05 (1,01‐1,10, *P* = 0,009)) y complicaciones (razón de oportunidades, odds ratio, OR 1,05 (1,01‐1,10, *P* = 0,009)).

**Conclusión:**

la NASBO ha puesto en evidencia los malos resultados en pacientes con SBO por hernia, subrayando la necesidad de iniciativas para mejorarlos.

## Introduction

Much of the literature addressing the treatment of small bowel obstruction (SBO) focuses on management of the most common cause, adhesions^1^, ^2^, ^3^. Emergency treatment of the obstructed abdominal wall hernia contains knowledge gaps that include the optimal surgical approach and type of repair^4^, ^5^. Care for this group is particularly challenging as patients are often elderly with high levels of co‐morbidity^6^.

The quality of emergency surgical care has been scrutinized through initiatives such as the American College of Surgeons' National Surgical Quality Improvement Program (NSQIP)^7^ and the UK National Emergency Laparotomy Audit^8^, although the latter study did not include many emergency repairs that failed to meet criteria for inclusion. UK evidence also suggests that ‘watchful waiting’ as an alternative to elective hernia repair has increased the rate of emergency procedures^9^.

The aim of this study was to analyse cohort data from the UK National Audit of Small Bowel Obstruction (NASBO) to describe the current management and outcomes of patients with SBO due to abdominal wall hernia.

## Methods

NASBO was a trainee‐led and trainee‐delivered multicentre prospective cohort study carried out between 16 January and 13 March 2017, with support from all major professional stakeholders. Any acute hospital in the UK that performed emergency general surgery was eligible to contribute data. Hospitals were recruited through the NASBO network, personal contacts and social media. This study is reported in line with STROBE^10^ and Statistical Analyses and Methods in the Published Literature (SAMPL)^11^ guidelines.

### Approvals

At each site, local collaborators were responsible for registering the study and securing Caldicott Guardian permissions. As this study was considered clinical audit, national research ethical approval was deemed not to be required (NHS East Scotland Research and Ethics Committee, NR/1610AB10).

### Inclusion and exclusion criteria

The study included non‐pregnant patients aged 16 years and over who had a clinical suspicion of SBO. Clinical suspicion was either identified by a member of the surgical team or agreed in consultation with other specialties. Patients subsequently found to have pathologies other than SBO were excluded.

### Data collection

Data were captured during the study period according to a prespecified protocol^12^ and entered into a secure REDCap (Research Electronic Data Capture) database hosted by the University of Sheffield. Fields collected were listed in the study protocol and included patient characteristics (age, sex, co‐morbidity as measured with Charlson Co‐morbidity Index^13^, risk of malnutrition, BMI, evidence of acute kidney injury and admission white cell count), imaging (use of abdominal plain film radiography, and CT), referring team or service, nutritional assessment and interventions (use of oral supplements and parenteral nutrition), management including surgery, and 30‐day in‐hospital outcomes including mortality (measured as survival), in‐hospital complications, length of stay and any requirement for readmission. In‐hospital complications included infectious complications (lower respiratory tract infection, superficial and deep surgical‐site infection and urinary tract infection), surgical complications (abdominal wall dehiscence and anastomotic leak), reintervention (radiological or reoperation), delirium, cardiovascular events, pulmonary embolism, deep venous thrombosis, and unplanned critical care admission.

Date of resumption of enteral nutrition was recorded by the local teams as the first date that oral intake was tolerated. Hernia types were broadly categorized as inguinal/femoral, primary midline, incisional and parastomal. The case report form and definitions of outcomes have been published previously^12^. Surgical interventions were classified as immediate operation (within 24 h of admission), delayed operation (24 h after admission) or non‐operative. Components of emergency procedures were also recorded, including small bowel resection and stoma formation.

### Data validation

Accuracy of data submission was validated by local investigators who were independent from data collection teams. Selected fields from 25 per cent of all submitted patient records were sampled randomly at each site. Data were re‐entered separately by the validator, who was blinded to the original data. Categorical variables were deemed to be accurate on exact match, and continuous variables when the value was within 0·5 units of the collected data. Accuracy is expressed as a percentage of correct fields of the total fields sampled.

### Statistical analysis

After exclusion of ineligible patients, data were summarized using simple descriptive statistics for comparisons across specific hernia groups. Data are presented as numbers and percentages for categorical data and as mean(s.d.) values for normally distributed continuous data; where data were not normally distributed, median (i.q.r.) values were used. χ^2^ and Kruskal–Wallis tests were used to test for differences between categorical and continuous variables respectively.

To adjust for confounding factors, clinically plausible variables were entered into a Cox proportional hazards model, clustered by centre to adjust for hospital‐level effects. Effect estimates are presented as hazard ratios (HRs) with corresponding 95 per cent confidence intervals. For the outcome of complications, a multilevel logistic regression model was used to estimate odds ratios (ORs) with 95 per cent confidence intervals. Briefly, patient risk factors were adjusted for at level 1 (fixed effect) and centre effects at level 2 (random effects). Model selection was guided by minimization of the Akaike information criterion. Models were examined for first‐level interactions, and those found to be significant were retained in the model. Statistical significance was taken at the level of *P* ≤ 0·050 *a priori*.

All analyses were performed in R version 3.4.4 (R Foundation for Statistical Computing, Vienna, Austria) using the tidyverse and finalfit packages.

## Results

**Fig 1:**
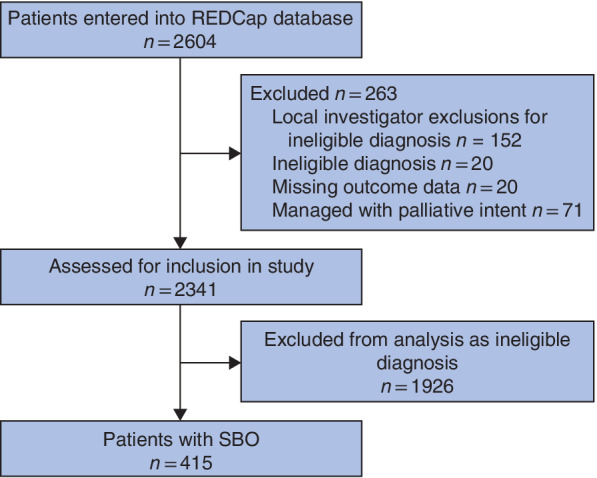
STROBE flow chart REDCap, Research Electronic Data Capture; SBO, small bowel obstruction.

NASBO collected data on 2604 patients from 131 hospitals during the study period (*Fig*. *1*). After screening, 2341 patients were assessed and included in the study analysis. In total, 415 patients (17·7 per cent) had SBO caused by abdominal wall hernia, making hernia the second most common cause of obstruction after adhesions (1150 patients, 49·1 per cent). Patients with hernia who were managed with end‐of‐life care were excluded from the analysis (10, 2·4 per cent). The data validation exercise demonstrated a high level of accuracy, with complete agreement between entered and validated patients at 92·4 per cent.

### Demographics

**Fig 2:**
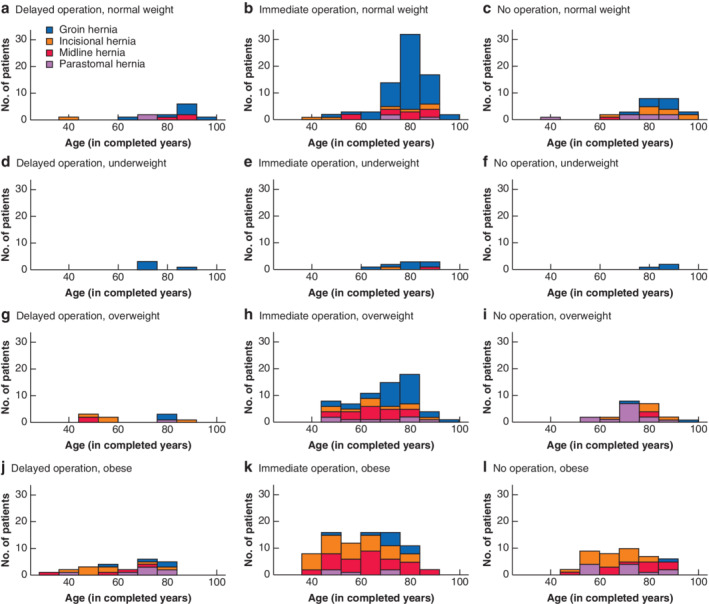
Histograms showing distribution of hernia type by age, BMI and treatment strategy

Patient characteristics are summarized in *Table* *1*. Those with a groin hernia were older than patients with other hernia types (mean(s.d.) age 78·3(10·4) *versus* 64·8(14·9) years respectively) (*Table* *1* and *Fig*. *2*). There were no differences in co‐morbidity amongst hernia groups.

**Table 1:** Patient characteristics

	Inguinal/femoral groin hernia (*n* = 153)	Midline hernia (*n* = 91)	Incisional hernia (*n* = 106)	Parastomal hernia (*n* = 65)	*P*†
Age at admission to study (years)*	78·3(10·4)	68·3(13·6)	64·8(14·9)	69·1(12·6)	< 0·001‡
**Sex ratio (M** : **F)**	66 : 87	50 : 41	38 : 68	34 : 31	0·032
CCI score*	4·6(7·4)	4·8(7·0)	4·4(6·8)	4·8(7·5)	0·747‡
**Nutritional Risk Index score**					0·468
Low	67 (43·8)	52 (57)	51 (48·1)	38 (58)	
Moderate	55 (35·9)	29 (32)	34 (32·1)	17 (26)	
Severe	6 (3·9)	2 (2)	5 (4·7)	1 (2)	
Missing	25 (16·3)	8 (9)	16 (15·1)	9 (14)	
**BMI**					< 0·001
Normal weight	71 (46·4)	14 (15)	15 (14·2)	12 (18)	
Underweight	14 (9·2)	1 (1)	1 (0·9)	0 (0)	
Overweight	33 (21·6)	21 (23)	19 (17·9)	22 (34)	
Obese	15 (9·8)	49 (54)	59 (55·7)	23 (35)	
Missing	20 (13·1)	6 (7)	12 (11·3)	8 (12)	
**Accommodation before admission**					0·180
Own home	143 (93·5)	87 (96)	102 (96·2)	64 (98)	
Residential home	5 (3·3)	0 (0)	0 (0·0)	0 (0)	
Nursing home	5 (3·3)	4 (4)	3 (2·8)	1 (2)	
Missing	0 (0)	0 (0)	1 (0·9)	0 (0)	
**Referral source**					0·097
Direct to surgical team	131 (85·6)	86 (95)	95 (89·6)	61 (94)	
From inpatient team	22 (14·4)	5 (5)	11 (10·4)	4 (6)	
**AKI on admission**					0·002
No	101 (66·0)	67 (74)	89 (84·0)	55 (85)	
Yes	52 (34·0)	24 (26)	17 (16·0)	10 (15)	
**WCC on admission (× 10** ^**9**^ **/l)**					0·448
≤ 11·9	90 (58·8)	46 (51)	60 (56·6)	29 (45)	
12·0–15·9	36 (23·5)	27 (30)	28 (26·4)	18 (28)	
≥ 16·0	27 (17·6)	18 (20)	18 (17·0)	18 (28)	
**Final treatment group**					< 0·001
No surgery	17 (11·1)	17 (19)	34 (32·1)	35 (54)	
Immediate operation	115 (75·2)	65 (71)	57 (53·8)	17 (26)	
Delayed operation	21 (13·7)	9 (10)	15 (14·2)	13 (20)	

Values in parentheses are percentages unless indicated otherwise; *values are mean(s.d.) CCI, Charlson Co‐morbidity Index; AKI, acute kidney injury; WCC, white cell count. †χ^2^ test, except ‡Kruskal–Wallis test.

Acute kidney injury was more prevalent in groin and primary midline hernias (34·0 and 26 per cent respectively) compared with incisional and parastomal hernia (16·0 and 15 per cent respectively) (*P* = 0·002).

More than one‐third of patients (149, 35·9 per cent) were at moderate to severe risk of malnutrition using the Nutritional Risk Index, which was highest in the groin and incisional hernia groups. Patients in the midline, incisional and parastomal hernia groups were significantly more likely to be overweight or obese as measured by BMI (77, 73·6 and 69 per cent respectively), in contrast with those in the groin hernia group, who tended to have normal BMI (31·4 per cent combined obese or overweight; 46·4 per cent normal BMI) (*Table* *1*).

### Imaging

Some 91 patients (21·9 per cent) had plain abdominal radiography only, and the remainder had abdominal CT with or without previous plain radiography. Patients with obstructed parastomal or incisional hernia underwent diagnostic CT in 86 and 84·0 per cent of cases respectively, compared with 71·9 per cent of patients with groin hernia and 66 per cent of those with midline hernia. Thirty‐five patients (11·1 per cent) had a subsequent water‐soluble contrast study to assess whether the hernia was obstructed. Nine patients (5 groin, 1 incisional, 3 midline) had no imaging performed to support the clinical diagnosis of obstruction (*Table* *2*).

### Operative interventions

Operative management was performed in 312 (75·2 per cent) of the 415 patients; *Table* *S1* (supporting information) summarizes the surgical procedures carried out. Immediate operation was more common in the groin hernia group at 75·2 per cent compared with midline (71 per cent), incisional (53·8 per cent) and parastomal (26 per cent) hernias (*Table* *S1*). In contrast, non‐operative management was initiated in 54 per cent of patients with parastomal hernia and 32·1 per cent of those with incisional hernia. Overall, small bowel resection was common, being required in 198 (63·5 per cent) of the 312 patients, and stoma formation was performed in 55 (17·3 per cent).

**Table 2:** Management

	Inguinal/femoral groin hernia (*n* = 153)	Midline hernia (*n* = 91)	Incisional hernia (*n* = 106)	Parastomal hernia (*n* = 65)	P*
**Radiology performed**					
No imaging	5 (3·3)	3 (3)	1 (0·9)	0 (0)	0·015
Abdominal X‐ray only	38 (24·8)	28 (31)	16 (15·1)	9 (14)	
CT only	28 (18·3)	9 (10)	21 (19·8)	7 (11)	
CT and abdominal X‐ray	82 (53·6)	51 (56)	68 (64·2)	49 (75)	
**Abdominal X‐ray**					
No	33 (21·6)	12 (13)	22 (20·8)	7 (11)	0·131
Yes	120 (78·4)	79 (87)	84 (79·2)	58 (89)	
**Diagnostic CT**					
No	43 (28·1)	31 (34)	17 (16·0)	9 (14)	0·003
Yes	110 (71·9)	60 (66)	89 (84·0)	56 (86)	
**Oral or rectal water‐soluble contrast agent except when having a scan?**					
No	142 (92·8)	86 (95)	96 (90·6)	56 (86)	0·269
Yes	11 (7·2)	5 (5)	10 (9·4)	9 (14)	
**Operative management**					
Non‐operative	17 (11·1)	17 (19)	34 (32·1)	35 (54)	< 0·001
Immediate operation	115 (75·2)	65 (71)	57 (53·8)	17 (26)	
Delayed operation	21 (13·7)	9 (10)	15 (14·2)	13 (20)	

Values in parentheses are percentages. *χ^2^ test.

### Nutritional intervention

The mean(s.d.) time without enteral nutrition was 6·8(8·9) days for groin hernia, 4·7(4·4) days for midline, 6·4(8·1) days for incisional and 7·7(13·0) days for parastomal hernia. Dietitian review was performed for 87 (63·5 per cent) of 137 patients who were identified as being at risk of malnutrition on screening. Nutritional intervention in the form of total parenteral nutrition was provided to 36 (41 per cent) of these 87 patients, at a mean(s.d.) of 11·3(16·9) days without enteral nutrition.

### Outcomes

The overall 30‐day in‐hospital mortality rate was 9·4 per cent (39 of 415), and was higher in patients with SBO due to obstructed groin hernia (17 of 153, 11·1 per cent) compared with other hernia types (*Table* *3*). Complications were common across all hernia subgroups, with lower respiratory tract infection occurring in 10·4–16·3 per cent, deep surgical‐site infection in 2–4·6 per cent, delirium in 3–11·1 per cent, and cardiovascular complications in 3–8·5 per cent (*Table* *3*). The mean(s.d.) length of hospital stay was 10·5(11·5) days. In patients who had surgery, the unadjusted mortality rate was 8·3 per cent (26 of 312), compared with 12·6 per cent (13 of 103) in the non‐operative group.

**Table 3:** Outcomes by hernia type

	**Inguinal/femoral groin hernia (*n* = 153)**	**Midline hernia (*n* = 91)**	**Incisional hernia (*n* = 106)**	**Parastomal hernia (*n* = 65)**	***P*†**
**30‐day in‐hospital death**					
No	136 (88·9)	84 (92)	97 (91·5)	59 (91)	0·815
Yes	17 (11·1)	7 (8)	9 (8·5)	6 (9)	
**Urinary tract infection**					
No	140 (91·5)	88 (97)	102 (96·2)	63 (97)	0·188
Yes, not urinary catheter‐associated	5 (3·3)	2 (2)	4 (3·8)	2 (3)	
Yes, urinary catheter‐associated	5 (3·3)	0 (0)	0 (0)	0 (0)	
Missing	3 (2·0)	1 (1)	0 (0)	0 (0)	
**Lower respiratory tract infection**					
No	126 (82·4)	79 (87)	95 (89·6)	55 (85)	0·612
Yes	25 (16·3)	11 (12)	11 (10·4)	10 (15)	
Missing	2 (1·3)	1 (1)	0 (0)	0 (0)	
**Deep SSI**					
No	144 (94·1)	86 (95)	102 (96·2)	64 (98)	0·754
Yes	7 (4·6)	4 (4)	4 (3·8)	1 (2)	
Missing	2 (1·3)	1 (1)	0 (0)	0 (0)	
**Superficial SSI**					
No	145 (94·8)	84 (92)	97 (91·5)	58 (89)	0·406
Yes	6 (3·9)	6 (7)	9 (8·5)	7 (11)	
Missing	2 (1·3)	1 (1)	0 (0)	0 (0)	
**Abdominal wall dehiscence**					
No	148 (96·7)	87 (96)	102 (96·2)	64 (98)	0·227
Yes	1 (0·7)	3 (3)	4 (3·8)	1 (2)	
Missing	4 (2·6)	1 (1)	0 (0)	0 (0)	
**Anastomotic leak**					
No	148 (96·7)	89 (98)	104 (98·1)	65 (100)	0·727
Yes	3 (2·0)	1 (1)	2 (1·9)	0 (0)	
Missing	2 (1·3)	1 (1)	0 (0)	0 (0)	
**Radiologically guided drainage**					
No	149 (97·4)	89 (98)	106 (100)	64 (98)	0·564
Yes	1 (0·7)	1 (1)	0 (0)	1 (2)	
Missing	3 (2·0)	1 (1)	0 (0)	0 (0)	
**Venous thromboembolism (PE or DVT)**					
No	145 (94·8)	89 (98)	106 (100·0)	65 (100)	0·146
Yes	5 (3·3)	1 (1)	0 (0)	0 (0)	
Missing	3 (2·0)	1 (1)	0 (0)	0 (0)	
**Delirium**					
No	134 (87·6)	84 (92)	100 (94·3)	63 (97)	0·265
Yes	17 (11·1)	6 (7)	6 (5·7)	2 (3)	
Missing	2 (1·3)	1 (1)	0 (0)	0 (0)	
**Cardiovascular event (MI, new heart block, stroke, TIA)**					
No	137 (89·5)	87 (96)	99 (93·4)	63 (97)	0·295
Yes	13 (8·5)	3 (3)	7 (6·6)	2 (3)	
Missing	3 (2·0)	1 (1)	0 (0)	0 (0)	
**Reoperation**					
No	129 (84·3)	70 (77)	67 (63·2)	28 (43)	< 0·001
Yes	5 (3·3)	3 (3)	5 (4·7)	2 (3)	
Missing	19 (12·4)	18 (20)	34 (32·1)	35 (54)	
**Unplanned critical care admission**					
No	134 (87·6)	82 (90)	97 (91·5)	60 (92)	0·708
Yes, ICU	6 (3·9)	4 (4)	4 (3·8)	4 (6)	
Yes, HDU	11 (7·2)	4 (4)	3 (2·8)	1 (2)	
Missing	2 (1·3)	1 (1)	2 (1·9)	0 (0)	
**Readmission within 30 days**					
No	126 (82·4)	78 (86)	84 (79·2)	53 (82)	0·310
Yes	21 (13·7)	9 (10)	20 (18·9)	12 (18)	
Missing	6 (3·9)	4 (4)	2 (1·9)	0 (0)	
Length of stay (days)*	10·9(10·8)	10·0(13·0)	9·3(10·1)	12·1(13·2)	0·505‡

Values in parentheses are percentages unless indicated otherwise;

* values are mean(s.d.). SSI, surgical‐site infection; PE, pulmonary embolism; DVT, deep venous thrombosis; MI, myocardial infarction; TIA, transient ischaemic attack; HDU, high‐dependency unit.

† χ^2^ test, except

‡ Kruskal–Wallis test.

### Modelling of outcomes

Cox proportional hazards modelling showed that delayed surgery was associated with lower mortality (adjusted HR 0·21, 95 per cent c.i. 0·06 to 0·80; *P* = 0·022) (*Table* *4*). Increased age was associated with increased hazards of mortality in all groups (adjusted HR 1·05 per unit increase, 95 per cent c.i. 1·01 to 1·10; *P* = 0·009). Small bowel resection was associated with decreased hazards of mortality in univariable analysis (HR 0·23, 0·09 to 0·57; *P* = 0·001), but this did not persist in multivariable modelling (*Table* *4*).

**Table 4:** Cox proportional hazards analysis of determinants of in‐hospital mortality (survival)

	**Univariable analysis**		**Multivariable analysis**	
	**Hazard ratio**	***P***	**Hazard ratio**	***P***
**Final treatment group**				
Non‐operative	1·00 (reference)		1·00 (reference)	
Immediate operation	0·46 (0·23, 0·93)	0·030	0·53 (0·20, 1·41)	0·202
Delayed operation	0·32 (0·11, 0·91)	0·032	0·21 (0·06, 0·80)	0·022
**Timing of CT**				
No CT	1·00 (reference)		1·00 (reference)	
< 24 h	0·92 (0·39, 2·16)	0·854	0·68 (0·29, 1·61)	0·382
24–48 h	0·58 (0·07, 4·72)	0·610	0·42 (0·03, 5·97)	0·524
> 48 h	0·83 (0·30, 2·33)	0·724	0·57 (0·17, 1·92)	0·361
**Age at admission to study**	1·06 (1·02, 1·09)	0·001	1·05 (1·01, 1·10)	0·009
**Sex**				
M	1·00 (reference)		1·00 (reference)	
F	1·01 (0·53, 1·92)	0·977	1·27 (0·58, 2·79)	0·554
**Charlson Co‐morbidity Index**	1·01 (0·97, 1·06)	0·489	0·99 (0·94, 1·04)	0·769
**Nutritional Risk Index score**				
Low	1·00 (reference)		1·00 (reference)	
Moderate	0·85 (0·42, 1·72)	0·653	0·76 (0·36, 1·63)	0·485
Severe	0·46 (0·06, 3·46)	0·451	0·32 (0·04, 2·71)	0·294
**WHO BMI class**				
Normal weight	1·00 (reference)		1·00 (reference)	
Underweight	0·67 (0·19, 2·33)	0·529	0·42 (0·10, 1·82)	0·245
Overweight	0·62 (0·26, 1·46)	0·276	0·75 (0·26, 2·15)	0·590
Obese	0·39 (0·16, 0·97)	0·042	0·57 (0·20, 1·61)	0·284
**Hernia type**				
Inguinal/femoral	1·00 (reference)		1·00 (reference)	
Other site	0·86 (0·46, 1·63)	0·645	1·01 (0·37, 2·74)	0·980
**Admission WCC (× 10** ^**9**^ **/l)**				
≤ 11·9	1·00 (reference)		1·00 (reference)	
12·0–15·9	1·24 (0·58, 2·67)	0·583	1·04 (0·35, 3·07)	0·941
≥ 16·0	1·31 (0·59, 2·87)	0·505	1·52 (0·58, 3·96)	0·395
**Initial management strategy**				
Non‐operative	1·00 (reference)			
Operative (decision within 24 h of admission)	0·74 (0·39, 1·38)	0·339		
**Referral source**				
Direct to surgical team (ED, GP, clinic)	1·00 (reference)		1·00 (reference)	
Other inpatient team	1·04 (0·47, 2·30)	0·916	1·13 (0·41, 3·16)	0·814
**AKI on admission**				
No	1·00 (reference)			
Yes	0·78 (0·38, 1·61)	0·503		
**Small bowel resection**				
No	1·00 (reference)			
Yes	0·23 (0·09, 0·57)	0·001		

Values in parentheses are 95 per cent confidence intervals. WCC, white cell count; ED, emergency department; GP, general practitioner; AKI, acute kidney injury.

Multilevel regression analysis to identify factors associated with major complications (death, unplanned high‐dependency unit/ICU admission, 30‐day readmission) indicated in a univariable model that operative management, increasing age, increasing co‐morbidity, CT within 24 h of admission or beyond 72 h after admission, and referral from other inpatient team were all significant predictors of complications, but only age persisted in the multivariable model (adjusted OR 1·05, 95 per cent c.i. 1·02 to 1·09; *P* = 0·001) (*Table* *5*).

**Table 5:** Multilevel logistic regression analysis of major complications

	Major complication*		Univariable analysis		Multivariable analysis	
	No (*n* = 346)	Yes (*n* = 66)	Odds ratio†	*P*	Odds ratio†	*P*
**Final treatment group**						
Non‐operative	89 (25·7)	13 (20)	1·00 (reference)		1·00 (reference)	
Immediate operation	214 (61·8)	39 (59)	1·25 (0·65, 2·53)	0·520	1·86 (0·82, 4·22)	0·139
Delayed operation	43 (12·4)	14 (21)	2·23 (0·96, 5·21)	0·061	2·64 (0·92, 7·57)	0·071
**Age at admission to study (years)‡**	69·9(14·2)	78·2(9·7)	1·06 (1·03, 1·08)	< 0·001	1·05 (1·02, 1·09)	0·001
**Sex**						
M	161 (46·5)	25 (38)	1·00 (reference)		1·00 (reference)	
F	185 (53·5)	41 (62)	1·43 (0·84, 2·48)	0·197	1·26 (0·66, 2·43)	0·484
**CCI score‡**	4·2(7·0)	6·4(8)	1·04 (1·00, 1·07)	0·028	1·04 (1·00, 1·08)	0·079
**WHO BMI class**	*n* = 312	*n* = 56				
Normal weight	91 (29·2)	21 (38)	1·00 (reference)		1·00 (reference)	
Underweight	12 (3·8)	4 (7)	1·44 (0·38, 4·63)	0·557	1·53 (0·41, 5·75)	0·527
Overweight	81 (26·0)	13 (23)	0·70 (0·32, 1·46)	0·345	1·15 (0·49, 2·71)	0·740
Obese	128 (41·0)	18 (32)	0·61 (0·30, 1·21)	0·156	1·26 (0·55, 2·91)	0·580
**Admission WCC (× 10** ^**9**^ **/l)**						
≤ 11·9	182 (52·6)	40 (61)	1·00 (reference)			
12·0–15·9	93 (26·9)	16 (24)	0·78 (0·41, 1·45)	0·447		
≥ 16·0	71 (20·5)	10 (15)	0·64 (0·29, 1·30)	0·242		
**Timing of CT**	*n* = 339					
No CT	91 (26·8)	9 (14)	1·00 (reference)			
< 24 h	198 (58·4)	42 (64)	2·14 (1·04, 4·87)	0·050		
24–48 h	14 (4·1)	2 (3)	1·44 (0·21, 6·37)	0·659		
> 48 h	36 (10·6)	13 (20)	3·65 (1·45, 9·57)	0·007		
**Hernia type**						
Inguinal/femoral	122 (35·3)	30 (45)	1·00 (reference)			
Other site	224 (64·7)	36 (55)	0·65 (0·38, 1·12)	0·117		
**Nutritional Risk Index score**	*n* = 300	*n* = 56				
Low	180 (60·0)	27 (48)	1·00 (reference)			
Moderate	109 (36·3)	26 (46)	1·59 (0·88, 2·87)	0·123		
Severe	11 (3·7)	3 (5)	1·82 (0·39, 6·28)	0·382		
**Referral source**						
Direct to surgical team (ED, GP, clinic)	316 (91·3)	54 (82)	1·00 (reference)			
Other inpatient team	30 (8·7)	12 (18)	2·34 (1·09, 4·76)	0·022		
**Initial management strategy**						
Non‐operative	132 (38·2)	27 (41)	1·00 (reference)			
Operative (decision within 24 h of admission)	214 (61·8)	39 (59)	0·89 (0·52, 1·54)	0·673		
**AKI on admission**						
No	265 (76·6)	44 (67)	1·00 (reference)			
Yes	81 (23·4)	22 (33)	1·64 (0·91, 2·87)	0·090		

Values in parentheses are *percentages and †95 per cent confidence intervals, unless indicated otherwise; ‡values are mean(s.d.). CCI, Charlson Co‐morbidity Index; WCC, white cell count; ED, emergency department; GP, general practitioner; AKI, acute kidney injury.

## Discussion

This study provides contemporary evidence of high rates of mortality and morbidity in patients with SBO due to obstructing hernia. Although the morbidity of obstructed hernia has been well documented^5^, this study demonstrates that it still persists.

In patients undergoing conservative treatment of groin hernia, the reported annual risk of strangulation or incarceration ranges from 0·4 to 2·7 per cent^14^. A recent study^15^ of emergency repaired midline hernia using the US NSQIP database showed the mortality rate to be 1·3 per cent, and a 2010 UK‐based single‐centre study^16^ found a 30‐day mortality rate after emergency groin hernia repair of 2·5 per cent. A single‐centre observational study^17^ of incarcerated hernia found morbidity comparable to that in the present study. These studies included a mix of obstructed and strangulated hernia, but the difference in mortality between these and the present study is stark. The finding that bowel resection in the present study might be associated with reduced mortality, as seen in *Table* *4*, contrasts with other studies^18^, suggesting that prompt surgery for clinically apparent bowel ischaemia improved outcomes. Equally, this difference may reflect selection bias, whereby fitter patients underwent resection and less fit patients were managed conservatively, with either hernia reduction alone or supportive conservative management. The association between delayed operation and lower in‐hospital mortality may reflect the predominantly incisional or parastomal hernia groups treated in this way. It might also reflect a group in which additional resuscitation measures had been undertaken, mitigating the impact of surgery.

The characteristics of the population studied may go some way to explaining the outcomes demonstrated here. Acute kidney injury was independently associated with poor outcomes, including death, in the present study, in line with other emergency surgeries^19^. Malnutrition is also associated with poor surgical outcomes. This study showed high rates of malnutrition with limited interventions to address this.

Beyond patient characteristics are those factors related to the organization of hospital services. Outcomes were worse in terms of complications for the patients referred from inpatient teams in this study, potentially reflecting delay where patients were admitted under medical specialties with ‘coffee ground vomit’ or ‘gastroenteritis’, whereas the correct diagnosis was obstructed hernia. Time to surgery may be one element of management that might be improved for this particular set of patients. However, it was noted that delayed surgery was associated with reduced mortality. This may reflect that the group who had delayed surgery were more likely to have incisional rather than groin hernias, the latter carrying a greater risk of strangulation.

Bowel resection rates were high in this study at 63·5 per cent, compared with 5 per cent in adhesive SBO^3^. Delay potentially increases the need for small bowel resection, which is associated with worse clinical outcomes including mortality^20^. A threshold of a 3‐day trial of non‐operative management has been demonstrated as the cut‐off point for worsening outcomes in adhesive obstruction^1^. It is possible that a similar temporal cut‐off exists for patients with obstructed hernia, albeit at a much earlier time point.

There were relatively high rates of unplanned critical care use (approximately 10 per cent) in the groin hernia population. Despite high levels of co‐morbidity, patients were managed at ward level until deterioration requiring increased support. In the UK, some of this patient group fell outside the National Emergency Laparotomy Audit criteria^7^, and were not assessed routinely in the same way for mortality risk and frailty. As a result, acuity of the situation and the need for critical care may not have been recognized for the high‐risk patient.

This study includes a subgroup of the whole patient population treated for SBO. As a result, it is missing some information that may be of interest to clinicians, specifically type of repair, use of mesh, and type of anaesthesia. As an observational study, no inference can be drawn on causality.

Guidelines for the management of hernia in the acute setting are limited, reflecting a paucity of high‐quality evidence^5^. Work in emergency laparotomy^8^, a condition with similar rates of mortality and morbidity, has demonstrated improvements in the UK. Principles learned from that study might be translated into emergency hernia surgery. For example, comprehensive preoperative risk assessment could inform preoperative and postoperative intervention. Mortality risk and frailty scoring could be undertaken^8^, ^21^. In turn, this information might influence case selection to avoid operating where futility is anticipated. Other researchers^9^, ^22^ have noted an increase in emergency hernia repair following the institution of watchful waiting policies, and the data presented here might shift the health economic balance of this policy to favour early repair in selected patients in the elective setting.

The findings of this study suggest that strategies should be developed to aid early diagnosis of hernia, particularly in patients admitted to non‐surgical wards, and that similar standards of care as offered to patients having emergency laparotomy (early imaging to aid diagnosis, senior surgeon, routine postoperative high‐dependency unit admission) should be instituted. To address these issues, additional data specific to emergency hernia repair, rather than only bowel obstruction, would be helpful. Prospective studies might investigate strategies to improve perioperative outcomes including preoperative resuscitation or the use of high‐dependency facilities. Decision‐making models to identify the optimal timing of repair for pre‐existing hernias might help surgeons to balance the risk of obstruction against the risks of repair in elective and emergency settings. Poor outcomes in patients with SBO due to abdominal wall hernia highlight the need for quality improvement initiatives in these patients.

## Supporting information


**Table S1** Procedures by hernia type
