# Supplementary material for: Outcomes of obstructed abdominal wall hernia: results from the UK national small bowel obstruction audit
Source: BJS Open. 2020 Jul 10;4(5):924–34. doi: 10.1002/bjs5.50315 (PMC7528520; doi:10.1002/bjs5.50315)
Supplement: Supplementary file 1 — Table S1 Procedures by hernia type [file BJS5-4-924-s001.docx]

**Appendix A**

**NASBO Steering Group**

Matthew J Lee, Thomas M Drake, Adele E Sayers, Ciaran J Walsh, Michael M Davies, Nicola S Fearnhead, John Abercrombie, Austin Acheson, Derek Alderson, Iain Anderson, Simon Bach, Michael Davies, Zaed Hamady, Daniel Hind, Marianne Hollyman, Sarah Hare, Ellen Lee, John Northover, Christopher Lewis, Paul Marriott, Nick Maynard, David Murray, Pritam Singh, Gillian Tierney, Azmina Verjee, Jonathan Wild

**NASBO Collaborators**

Abbott S, Abdulaal Y, Afshar S, Ah-Chuen J, Ahmed T, Akhtar M, Akram F, Aldred E, Ali A, Aly M, Amajuoyi A, Amin V, Anderson D, Anderson O, Andreou A, Ansari A, Appleton S, Ardley R, Arshad F, Ashour O, Asour A, Athem A, Athersmith M, Ayoub F, Azeem H, Azhar B, Badenoch T, Baillie C, Bandyopadhyay D, Barker J, Barker S, Barkham B, Baron R, Barrie J, Barry-Yarrow E, Bashir G, Battersby N, Bazoua G, Behar N, Bellam S, Berger C, Bhandari S, Bhasin S, Biggs S, Bisset C, Blake L, Blencowe N, Boam T, Boddy A, Boereboom C, Bogdan M, Bogle R, Bohra P, Boland M, Bolkan H, Borg C, Boulton R, Bouras G, Boyer M, Boyle J, Branagan G, Brewer H, Briggs C, Broadhurst J, Brown E, Brown J, Brown L, Brown O, Burns K, Butcher K, Butler M, Byrne B, Campbell L, Capper C, Cartmell M, Cash T, Chan S, Chandratreya N, Chapman J, Chapman S, Charalabopoulos A, Cheek C, Chok S, Choong W, Chow M, Chowdhury J, Coe P, Conaghan P, Conn G, Cook N, Cook T, Cooper S, Cornish J, Cotton D, Cox C, Coyne P, Crook R, Crozier J, Cuffolo G, Cunha P, Curtis N, Cutting J, Da Costa K, da Silva L, Das B, Davenport M, Davies J, Davies T, Day A, Dayal S, Dean S, Demetriou G, Dengu F, Dennis R, Dent H, Dent P, Deputy M, Devoto L, Di Benedetto G, Dindyal S, Donnelly E, Doody P, Douka E, Downham C, Dowson H, Edent H, Edgerton K, Ekpete N, El Farran M, Elamin O, Eljaafari M, Elsaid N, El-Sharif M, Evans J, Evans M, Ewe R, Ewing A, Exarchou K, Fallaize R, Faoury M, Farag S, Farinella E, Faulkner G, Ferguson H, Fisher O, Fletcher J, Forouzanfar A, Foster A, Fox R, Francis N, Fretwell V, Fung D, Gammeri E, Garnham J, Geraghty A, Gilbert A, Gill C, Gill M, Gillespie M, Giordano P, Glasbey J, Goh M, Golder A, Green N, Gregoir T, Grey T, Groundwater E, Grove T, Growcott S, Gunasekaran S, Habib H, Haddow J, Halahakoon V, Halkias C, Hall C, Hampson A, Hancock L, Hanna T, Hannay J, Harikrishnan A, Harries R, Harris G, Hartley J, Harvey K, Hawkin P, Hawkins J, Healy R, Heard R, Heartshorne R, Heller S, Hendra L, Herrod P, Heywood N, Hicks G, Hobson B, Holtham S, Holtham S, Hope C, Hopley P, Hossain T, Hossaini S, Howse F, Hubbard T, Humphreys A, Ikram H, Ioannis M, Iqbal M, Iqbal N, Jain R, Jatania J, Jenkinson P, Jokhan S, Jones A, Jones C, Jones L, Joshi H, Joshi K, Joy M, Jull P, Kakaniaris G, Kakaniaris G, Kallam R, Kane E, Kang P, Kanitkar R, Kauser S, Kazmi F, Kedrzycki M, Kelly S, Kendall J, Khan M, Khan T, King G, Kisiel A, Kitsis C, Kolawole I, Korambayil S, Kosasih S, Kosti A, Kotb A, Kouris S, Kshatriya K, Kumar S, Lafaurie G, Lal R, Lau A, Lazim T, Lazim T, Lazzaro A, Lee K, Lefroy R, Leinhardt D, Leinhardt D, Lennon H, Leong K, Levy B, Lim E, Lim J, Lindley S, Liu D, Lloyd P, Locker D, Lockwood S, Lowe C, Lund J, Lunevicius R, Lunt A, Lutfi S, Luther A, Luwemba S, Mahankali-Rao P, Mahroof S, Mai D, Majid S, Malik A, Malik K, Mann K, Mansour S, Manu N, Mapara R, Martin C, Martin J, Martin R, Mason C, Massey L, Mathias J, Mathur P, Maude K, McArthur D, McCain S, McCluney S, McFall M, McIlroy B, McKay S, McKinley N, McNair A, McWhirter D, Mekhail P, Mellor K, Merchant J, Merker L, Messenger D, Miles A, Mir S, Mishra A, Mistry P, Miu V, Moat M, Mockford K, Mohamed E, Mohamed I, Mondragon-Pritchard M, Moore N, Moretti L, Morris H, Morrison T, Morrison-Jones V, Moss J, Moug S , Mountford D, Moynihan R, Muhammad K, Muldoon-Smith D, Mulholland J, Mullan M, Murgitroyd E, Murugaiyan K, Myers A, Mykoniatis I, Nana G, Nash T, Nassar A, Newton R, Ng C, Ng P, Ng P, Nguyen K, Nguyen K, Nicholas F, Noor M, Nowers J, Nugent C, Nunn A, Nunn R, Obeid N, O'Callaghan J, O'Hara R, Oke O, Olivier J, O'Neill A, O'Neill S, Osei-Bordom D, Osgood L, Panagiotopoulos S, Panchasara B, Parks R, Patel H, Patel P, Patel R, Patel S, Pawelec K, Payne C, Pearson K, Perin G, Peristerakis I, Petronio B, Phelan L, Phillips J, Pisaneschi C, Pitt J, Plunkett-Reed K, Ponchietti L, Pouzi A, Pouzi M, Powell A, Powell-Chandler A, Pranesh N, Proctor V, Pywell S, Qureshi A, Qureshi N, Rahman M, Rai Z, Ramcharan S, Rangarajan K, Rashid M, Reader H, Rehman A, Rehman S, Rengifo C, Richards E, Richardson N, Robinson A, Robinson D, Rossi B, Rutherford F, Sadien I, Saghir T, Sahnan K, Salahia G, Sarveswaran J, Saunders M, Scott B, Scott K, Seager A, Seal S, Sezen E, Shaban F, Shah P, Shah P, Shahmohammadi M, Shamsiddinova A, Shankar S, Sharpe A, Shatkar V, Sheel A, Shields T, Shinkwin M, Shurmer J, Siddika A, Siddiqui S, Simson R, Sinclair P, Singh B, Singh S, Sivaraj J, Skaife P, Skelly B, Skinner A, Slim N, Smart C, Smart N, Smith F, Smith I, Smith R, Spence G, Sreedhar A, Steinke J, Stevenson L, Stewart-Parker E, Stott M, Stubbs B, Stubbs B, Stylianides N, Subramonia S, Swinkin M, Swinscoe M, Symons N, Tahir W, Taj T, Takacs K, Tam J, Tan K, Tani S, Tanner N, Tao D, Taylor M, Thava B, Thippeswamy K, Thomas C, Thompson E, Thompson R, Thompson-Reil C, Thorn C, Tongo F, Toth G, Turnbull A, Turnbull J, Valero C, van Boxel G, Varcada M, Venn M , Ventham N, Venza M, Vimalachandran D, Virlos I, Wade T, Wafi A, Waite K, Walker M, Walker N, Walker T, Walsh U, Wardle S, Warner R, Watfah J, Watson N, Watt J, Watts J, Wayman J, Weegenaar C, West H, West M, Whitehurst L, Whyler M, Wiggans M, Wijeyekoon S, Williams G, Williams R, Williamson A, Williamson J, Wilson J, Winter A, Wolpert L, Wong J, Yeap E, Yeong T, Zaman S, Zappa B, Zosimas D, West Midlands Research Collaborative.
